# VEB-1 in *Achromobacter xylosoxidans* from Cystic Fibrosis Patient, France

**DOI:** 10.3201/eid1211.060143

**Published:** 2006-11

**Authors:** Catherine Neuwirth, Carine Freby, Agathe Ogier-Desserrey, Stéphanie Perez-Martin, Anne Houzel, André Péchinot, Jean-Marie Duez, Fréderic Huet, Eliane Siebor

**Affiliations:** * Hôpital Universitaire du Bocage, Dijon, France

**Keywords:** Extended-spectrum beta-lactamase VEB-1, cystic fibrosis, Achromobacter xylosoxidans, integron, dispatch

## Abstract

Multidrug-resistant *Achromobacter xylosoxidans* was recovered from the sputum of a patient with cystic fibrosis. The VEB-1 extended-spectrum β-lactamase was detected on a class 1 integron. This first report of a VEB-1–producing isolate in this population requires further investigation to determine its distribution.

Achromobacter (formerly Alcaligenes) xylosoxidans is a newly emerging microorganism isolated with increased frequency from the lungs of patients with cystic fibrosis (CF), but information about its clinical relevance is limited (*1*). A. xylosoxidans is innately resistant to many antimicrobial drugs (*2*), except piperacillin, piperacillin-tazobactam, and imipenem, and moderately susceptible to ceftazidime (45% of susceptible isolates), which is widely used to treat infection due to Pseudomonas aeruginosa (*3**,**4*). The mechanisms involved in cases of high-level resistance to ceftazidime have not been described for A. xylosoxidans. Possible mechanisms for ceftazidime resistance among gram-negative bacilli are alterations in outer membrane proteins, overproduction of cephalosporinase, or production of an extended-spectrum β-lactamase (ESBL). ESBLs are enzymes distributed worldwide (*5*) that hydrolyze oxyimino-cephalosporins and monobactams and are susceptible to β-lactamase inhibitors such as clavulanic acid and tazobactam. We report on the isolation from a CF patient of A. xylosoxidans that produced the VEB-1 ESBL. This is the first report of ESBL production in A. xylosoxidans and the first report of a VEB-1–producing isolate from a CF patient.

## The Study

During the past 10 years in our 1,600-bed university hospital, 37 CF patients had >1 respiratory tract specimen that contained A. xylosoxidans. Preliminary pulsed-field gel electrophoresis of these strains has failed to identify shared isolates among the patients, but studies are ongoing. In November 2003, A. xylosoxidans 476 (AX476) was isolated from the sputum of a 17-year-old male CF patient. This patient had good pulmonary function (forced expiratory volume 1 = 99% of predicted value), had never been colonized or infected by P. aeruginosa, and therefore never received ceftazidime. The strain was identified with the Api 20NE system (bio-Mérieux, Marcy-l'Etoile, France), and antimicrobial susceptibility testing was performed and interpreted as recommended by the Clinical and Laboratory Standards Institute (formerly NCCLS) (*6*).

The antibiogram, which was performed by a disk diffusion method, showed AX476 to be highly resistant to ceftazidime, aminoglycosides, sulfonamides, trimethoprim, and ciprofloxacin but fully susceptible to tetracyclines, piperacillin/tazobactam, ticarcillin/clavulanic acid, and imipenem. Because of an unusual synergy between ticarcillin and ticarcillin/clavulanic acid (Figure), we compared the inhibition zones of third-generation cephalosporin disks with and without clavulanic acid (BioRad, Marnes-la-Coquette, France). The zones were 7 mm for ceftazidime and 19 mm for ceftazidime plus clavulanic acid (Figure), which strongly indicated production of an ESBL. Isoelectric focusing showed that AX476 produced a β-lactamase with an isoelectric point of 7.4. A large plasmid of ≈200 kb (pJDB1) was easily transferred by conjugation to Escherichia coli K-12 C600. The transconjugants, E. coli (pJDB1) sorbitol-fermenting, which were selected on MacConkey agar that contained 4 μg/mL of ceftazidime, were resistant to sulfonamides and trimethoprim, had reduced susceptibility to aminoglycosides, and harbored a β-lactamase with an isoelectric point of 7.4. The resistance phenotype of the isolate and the value of the isoelectric point of the enzyme suggested the production of the ESBL VEB-1 (*7*).

**Figure:**
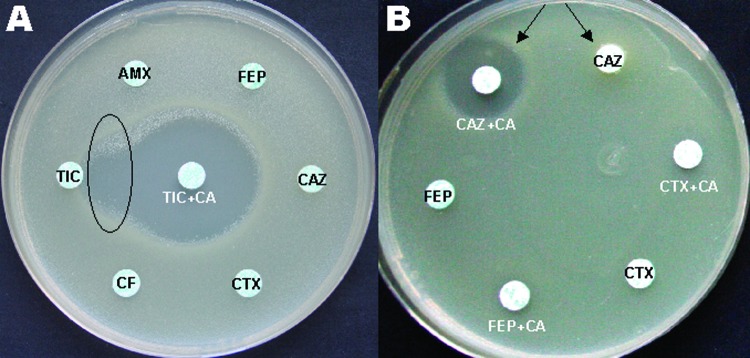
A) Oval indicates area of synergy between ticarcillin (TIC) and TIC plus clavulanic acid (CA). B) Arrows point to inhibition zone around third-generation disks with and without CA. AMX, amoxicillin; FEP, cefepime; CAZ, ceftazidime; CTX, cefotaxime; CF, cefalotin.

The MICs for β-lactams for AX476 and its transconjugant, determined by Mueller-Hinton broth dilution method, are shown in Table 1. By using bla_VEB-1_-specific primers, a positive PCR result was obtained on total DNA from AX476 and the transconjugants. All genetic analyses of bla_VEB-1_ published so far have identified either its chromosome (*8*) or its plasmid (*9*) location and mostly its integration within class 1 integrons of variable structure. Integrons are potentially mobile genetic elements that comprise conserved sequences that flank a variable region and may contain inserted antimicrobial drug resistance gene cassettes (*10*). The 5´-conserved segment includes the gene intI1 that encodes an integrase, the cassette integration site attI1, and a promoter responsible for the expression of the genes located downstream within the variable region. The 3´-conserved region contains either a qacEΔ1 gene that encodes resistance to quaternary ammonium compounds or a combination of 3 genes: qacEΔ1, sulI (which encodes resistance to sulfonamides), and orf5 (an open reading frame of unknown function).

**Table 1:** Beta-lactam MICs (μg/mL)

β-lactams*	*Achromobacter xylosoxidans* AX476	*Escherichia coli* K-12 C600 (pJDB1)†	*E. coli* K-12 C600‡
Amoxicillin	1,024	16	16
Amoxicillin + CA	32	2	8
Ticarcillin	256	64	8
Ticarcillin + CA	8	2	8
Cefotaxime	>512	0.06	0.03
Cefotaxime + CA	256	0.03	0.03
Ceftazidime	512	2	0.125
Ceftazidime + CA	16	0.125	0.125
Aztreonam	>512	4	0.125
Aztreonal + CA	256	0.125	0.125
Cefepime	512	0.06	0.03
Cefepime + CA	128	0.03	0.03

*CA, clavulanic acid, used at 2 μg/mL. †Transconjugant. ‡Reference strain.

To search for the presence of such a class 1 integron in AX476 and its transconjugant, we performed PCR on total DNA of AX476 and E. coli (pJDB1) by using the primers L1 and R1 specific for the detection of class 1 integrons (*11*). We obtained a fragment of 2.3 kb in the clinical strain and its transconjugant, which was sequenced on both strands. By using a set of primers, we deduced the structure of this integron (Table 2). Three gene cassettes have been identified. The first, dhfr (dihydrofolate reductase), encoded a putative trimethoprim-resistance protein. This dhfr was identical to that reported in Salmonella enterica serovar Typhi (GenBank accession no. AL513383) and to the dhfr gene cassette contained in a class 1 integron from Klebsiella pneumoniae not yet published (GenBank accession no. AJ971342). The second cassette, bla_VEB-1_, encoded the ESBL VEB-1 first described in E. coli (*7*). The third and last gene cassette was aadB. It encoded an aminoglycoside adenyltransferase that conferred resistance to kanamycin, gentamicin, and tobramycin and was identical to other sequenced aadB gene cassettes located on integrons containing bla_VEB-1_ gene (*7**,**8*). VEB-1 has been detected in Enterobacteriaceae and P. aeruginosa isolates from Southeast Asia (*9*) but never in A. xylosoxidans. In France, VEB-1–producing isolates of Acinetobacter baumannii have been involved in several outbreaks of nosocomial infection in intensive care units (*12**,**13*); however, we have not yet detected a VEB-1–producing isolate in our hospital.

**Table 2:** Primers used for PCRs

Amplified DNA	Primer	Oligonucleotide sequence (5´→3´)	GenBank accession no.
Variable region of class 1 integrons	L1	GGCATCCAAGCAGCAAGC	U49101
	R1	AAGCAGACTTGACCTGAT	U49101
*intI1*	Int-IN	TGTCGTTTTCAGAAGACGG	U49101
	IntA-R	ATCATCGTCGTAGAGACG	U49101
	IntB-F	GTCAAGGTTCTGGACCAG	U49101
*bla_VEB-1_*	VEB-R	GACTCTGCAACAAATACGC	AF010416
	VEB-outF	CAGCAGCCACTAATGATG	AF010416
	VEB-F	CCAGATAGGAGTACAGAC	AF010416
3´CS region	Qac-F	TCGCAATAGTTGGCGAAG	U49101
	Sul-F	GACGGTGTTCGGCATTCT	U49101
	Sul-R	TGAAGGTTCGACAGCACG	U49101
	Orf5-R	GATTTCGAGTTCTAGGCG	U49101
	Orf5-F	GGTGATATCGACGAGGTT	U49101

## Conclusions

This finding of a VEB-1–producing A. xylosoxidans from a CF patient enhances the scant information available to laboratorians and clinicians about ESBL production by isolates from CF patients. A very recent study reports 3 ESBL-positive isolates of P. aeruginosa from CF patients in New Delhi, but the ESBL has not been characterized (*14*). Resistance to expanded-spectrum cephalosporins mediated by ESBLs has never been described in A. xylosoxidans. The detection of the ESBL production was difficult in AX476; therefore, the frequency of A. xylosoxidans isolates that produce an ESBL might be underestimated. We recommend the use of BioRad combination disks, especially for isolates that are highly resistant to ceftazidime and susceptible to piperacillin or when synergy exists between ticarcillin and ticarcillin plus clavulanic acid.

The origin of the strain remains unclear. Because A. xylosoxidans is widely encountered in the environment, acquisition of AX476 by our patient may have resulted from poor adherence to handwashing, contamination of respiratory therapy equipment (nebulizer), or contaminated water. We can exclude nosocomial acquisition because our patient had never been hospitalized.

The location of bla_VEB-1_ on an easily transferable plasmid might represent a clinical threat if spread among other species widely encountered among CF patients, especially P. aeruginosa. Such a transfer would create serious therapeutic problems. Therefore, to prevent person-to-person transmission, our patient visits the physician on different days than the other CF patients. If he needs to be hospitalized, our patient may not share a room with immunocompromised patients or with other CF patients anywhere in the hospital, which is the recommendation for patients with other multidrug-resistant pathogens (*15*). In conclusion, this first finding of a VEB-1–producing A. xylosoxidans from a CF patient emphasizes the need to study the mechanism(s) of resistance to ceftazidime among a wide collection of isolates originated from different centers. The sequence of the class 1 integron reported in this paper has been assigned GenBank accession no. DQ393569.
